# Co-conjugation of Virulence Plasmid and KPC Plasmid in a Clinical *Klebsiella pneumoniae* Strain

**DOI:** 10.3389/fmicb.2021.739461

**Published:** 2021-11-08

**Authors:** Xuemei Yang, Ning Dong, Xiaoxuan Liu, Chen Yang, Lianwei Ye, Edward Wai-Chi Chan, Rong Zhang, Sheng Chen

**Affiliations:** ^1^Department of Infectious Diseases and Public Health, Jockey Club College of Veterinary Medicine and Life Sciences, City University of Hong Kong, Kowloon, Hong Kong SAR, China; ^2^State Key Lab of Chemical Biology and Drug Discovery, Department of Applied Biology and Chemical Technology, The Hong Kong Polytechnic University, Kowloon, Hong Kong SAR, China; ^3^Department of Clinical Laboratory, School of Medicine, Second Affiliated Hospital of Zhejiang University, Hangzhou, China

**Keywords:** *Klebsiella pneumonia*, conjugative virulence plasmid, *bla*_*KPC*_-bearing plasmid, transmission, CR-HvKP, IncFIB/IncHI1B, IncFII/IncR

## Abstract

Carbapenem-resistant hypervirulent *Klebsiella pneumoniae* (CR-HvKP) strains have been increasingly reported, and it is important to understand the evolutionary mechanisms of these highly pathogenic and resistant bacterial pathogens. In this study, we characterized a ST11 carbapenem-resistant *K. pneumoniae* strain which harbored an IncFIB/IncHI1B type virulence plasmid and an IncFII/IncR type *bla*_*KPC*__–__2_-bearing plasmid. The virulence plasmid was found to be conjugative and harbored a 35-kbp fragment including aerobactin encoding cluster from virulence plasmid pLVPK and multiple resistance genes, resulting in a mosaic multi-drug resistance and virulence plasmid. This virulence plasmid could be transferred via conjugation to *Escherichia coli* and *K. pneumoniae* strains alone as well as together with the *bla*_*KPC*__–__2_-bearing plasmid. Co-transmission of virulence and *bla*_*KPC*__–__2_-bearing plasmids would directly convert a classic *K. pneumoniae* strain into CR-HvKP strain, leading to a sharp increase in the prevalence of CR-HvKP in clinical settings, which poses a great threat to human health.

## Introduction

*Klebsiella pneumoniae* is a Gram-negative pathogen that has become a major causative agent of hospital infections worldwide in recent years (Paczosa and Mecsas, 2016; Grundmann et al., 2017; Gu et al., 2018). *K. pneumoniae* could acquire an accessory genome which makes the strain either hypervirulent or antibiotic resistant (Lin et al., 2008; Yang et al., 2020). The hypervirulent *K. pneumoniae* (hvKP) pathotype is undergoing global dissemination and was found to be highly associated with genes *peg-344*, *iroB*, *iucA*, *rmpA*, and *rmpA2*, all of which were typically encoded by a pLVPK-like virulence plasmid (Russo et al., 2018). These genes are involved in the aerobactin (Iuc) and salmochelin (Iro) siderophores synthesis and the upregulation of capsule production. The best characterized virulence plasmids are plasmid pLVPK recovered from K2 hvKP strain CG43 and plasmid pK2044 from K1 hvKP strain NTUH-K2044 which shared 96% coverage and 99% identity (Chen et al., 2004; Wu et al., 2009). Another well-characterized K1 hvKP strain, SGH10, was also found to harbor a 231,583-bp virulence plasmid, similar to plasmids pK2044 and pLVPK (Lam et al., 2018). Plasmid recombination between such virulence plasmid and other plasmids is increasingly being observed, resulting in mosaic plasmids encoding both virulence and resistance elements or conjugative virulence plasmids (Lam et al., 2019; Turton et al., 2019; Yang et al., 2019). Such genetic changes during the transmission process indicates that virulence plasmids have rapidly adapted to co-exist with *K. pneumoniae* strains (Lev et al., 2018; Russo et al., 2018). In this study, we characterized a CRKP strain which harbored a conjugative virulence and resistance co-encoding plasmid. Such plasmid was found to be able to conjugate to *Escherichia coli* and *K. pneumoniae* strains together with the KPC plasmid.

## Materials and Methods

### Bacterial Strains

*K. pneumoniae* strain SH12 was recovered from a clinical specimen from a patient at a hospital located in Shanghai in 2015 in a nationwide surveillance project in mainland China. This strain was isolated from a sputum sample of the hospitalized patient, who was diagnosed with pneumonia. A string test was performed on blood agar as previously described (Gu et al., 2018). *K. pneumoniae* strains CR-HvKP4 and FJ8 were reported in our previous study (Gu et al., 2018). Ciprofloxacin-resistant *K. pneumoniae* strain PM23 was a clinical isolate in Hong Kong provided by Prof. Kwok-yung Yuen from The University of Hong Kong. *E. coli* strains J53 and ATCC 25922 were from our laboratory stock.

### Antibiotic Susceptibility Testing

Antimicrobial susceptibility testing was performed by the micro dilution method. *E. coli* strain ATCC 25922 served as quality control strain for susceptibility testing. Antimicrobial agents including aztreonam, cefotaxime, ceftazidime, meropenem, imipenem, ertapenem, ciprofloxacin, amikacin, polymyxin B, tigecycline, ceftazidime-avibactam, and cefiderocol were tested. All tests were performed in duplicate, and each test included three biological replicates per strain. The susceptibility was interpreted according to both Performance Standards for Antimicrobial Susceptibility Testing by the Clinical and Laboratory Standards Institute (CLSI) of 2021 (CLSI, 2021) and Clinical Breakpoints and Guidance by European Committee on Antimicrobial Susceptibility Testing (EUCAST) of 2021 (The European Committee on Antimicrobial Susceptibility Testing, 2021).

### DNA Sequencing and Bioinformatics

Genomic DNA of strain SH12 was extracted using the PureLink Genomic DNA Mini Kit (Invitrogen, United States) according to the manufacturer’s instructions. The extracted DNA was then subjected to library preparation by NEBNext Ultra II DNA Library Prep Kit for Illumina (New England Biolabs, United States) and sequenced via the 150-bp paired-end NextSeq 500 platform (Illumina, San Diego, CA). Genomic DNA was also subjected to the long-read MinION platform (Oxford Nanopore Technologies, Oxford, United Kingdom). MinION libraries were prepared using the SQK-RBK004 nanopore sequencing kit according to the manufacturer’s instructions. The library was then added to a MinION flow cell (R9. 4.1) and sequenced. Base calling was performed using Guppy v5.0.11. Read trimming and filtering was performed using NanoFilt v2.8.0 (De Coster et al., 2018). Both short and long reads were *de novo* hybrid assembled using Unicycler v0.4.7 (Wick et al., 2017). Assembled genome sequences were annotated with RAST v2.0 (Brettin et al., 2015). Multi-locus sequence typing (MLST) were determined by the Kleborate software based on genetic variation in the seven housekeeping genes (Lam et al., 2020). Capsular typing and O-antigen typing on the assembled sequences were performed using Kaptive (Wyres et al., 2016). Virulence genes were identified by searching against the BIGSdb Klebsiella genome database.^1^ The BLAST command lines, with an 80% coverage and identity cutoff, were used to map genome sequences against antibiotic resistance genes and plasmid replicons. The resistance genes and plasmid replicons databases were obtained from the Center for Genomic Epidemiology.^2^ The insertion sequences (ISs) were identified using Isfinder and ISsaga^3^ (Siguier et al., 2006). Alignment of plasmids with similar structures were generated by Easyfig win_2.1 (Sullivan et al., 2011).

### Conjugation Assay

To determine the transferability of the virulence plasmid and KPC plasmid of strain SH12, conjugation was performed using azide-resistant *E. coli* strain J53 as recipient. Transfer of the plasmids from the donor strain to the recipient strain was determined by plating samples of a mating mixture onto selective media as previously described with modifications (Yang et al., 2019). As the virulence plasmid carried resistance gene *bla*_*DHA*__–__1_, the transconjugants carrying the virulence plasmid were selected by spreading the culture on eosin-methylene blue (EMB) agar plates containing 2 μg/ml cefotaxime and 100 μg/ml sodium azide. As transconjugants carrying the KPC plasmid can also grow on the EMB agar plates containing 2 μg/ml cefotaxime and 100 μg/ml sodium azide, colonies were further tested for growth on EMB plates containing 2 μg/ml meropenem. Those that could grow on both plates containing 2 μg/ml cefotaxime and 2 μg/ml meropenem were treated as KPC plasmid transconjugants, and those that could grow on plates containing 2 μg/ml cefotaxime rather than plates containing 2 μg/ml meropenem were treated as virulence plasmid transconjugants. The presence of *rmpA2* and *bla_KPC–2_* genes as marker genes of virulence plasmid and KPC plasmid in transconjugants was determined by polymerase chain reaction (PCR). The minimum inhibitory concentration (MIC) profiles of transconjugants were also determined to differentiate from the donor. Successful virulence plasmid transconjugants of *E. coli* strain J53 were then treated as donor and the ciprofloxacin-resistant *K. pneumoniae* strain PM23 as recipient. MacConkey agar plates containing 2 μg/ml cefotaxime together with 2 μg/ml ciprofloxacin were used to select transconjugants. S1 nuclease pulsed-field gel electrophoresis (S1-PFGE) was performed to confirm the transfer of plasmids. To calculate the conjugation efficiency, the diluted culture after conjugation was also plated on plates containing only 100 μg/ml sodium azide for strain J53 as recipient and 2 μg/ml ciprofloxacin for strain PM23 as recipient to count the total recipient cells, respectively. The conjugation efficiency was calculated as the number of transconjugants cells divided by the number of recipient cells. The conjugation efficiency was determined for transconjugants carrying virulence plasmid only, *bla*_*KPC*__–__2_-bearing plasmid only, and both plasmids.

### Plasmid Curing

Curing of the large virulence plasmid of strain SH12, pSH12_Vir, was performed using sodium dodecyl sulfate (SDS) (El-Mansi et al., 2000). Briefly, an SDS solution (10% w/v, pH 7.4) was added to Luria broth (LB) to give 5, 4, 3, 2, 1, and 0.5% SDS. An inoculum of 100 μl overnight-cultured strain SH12 was then used to seed the SDS-containing LB, and the cultures were incubated overnight with shaking at 27°C. After SDS treatment, the diluted culture of each concentration was spread on LB agar plates, respectively, to select virulence plasmid cured colonies. About 50 colonies from each concentration were passed on LB agar plates and tested for the presence of *rmpA2* and *iucA* genes, and those which proved negative were treated as plasmid cured strains. The plasmid-curing rate for the plasmid is 5.00E–03. Plasmid curing was confirmed by S1-PFGE analysis.

### Mouse Infection Model

Mouse bacteremia model was used to test the potential virulence of *K. pneumoniae* strain SH12. In this experiment, eight male Crl:CD1(ICR) mice in each group were infected intraperitoneally with an inoculum of 2.0 × 10^5^ and 1.0 × 10^6^ CFUs of different strains of *K. pneumoniae*, respectively. The mortality rate of the test mice was observed and recorded for 1 week post infection. Carbapenem-resistant, hypervirulent *K. pneumoniae* strain CR-HvKP4 reported in our previous study was used as control for high virulence, while CRKP strain FJ8 was used as control for low virulence (Gu et al., 2018; Yang et al., 2019). Strain CR-HvKP4 was a ST11 CRKP strain that harbored a pLVPK-like virulence plasmid, exhibiting hyper virulence. FJ8 was a classical CRKP strain which exhibited low virulence. All animal experiments were approved by the Animal Research Ethics Sub-Committee, City University of Hong Kong. Animal experiments were repeated twice to assess the consistency of the data.

## Results and Discussion

A strain suspected to be *K. pneumoniae*, SH12, was recovered from a sputum sample of a hospitalized patient in 2015 in Shanghai, China. Results of string test indicated that strain SH12 was string test negative. Antimicrobial susceptibility testing performed on SH12 showed that it was resistant to all β-lactam antibiotics, amikacin, and ciprofloxacin but remained susceptible to polymyxin B and ceftazidime-avibactam according to both CLSI and EUCAST standards and resistant to cefiderocol but susceptible to tigecycline according to EUCAST standards (Table 1).

**TABLE 1:** Phenotypic and genotypic characteristics of K. pneumoniae strain SH12 and its transconjugants.

**Strain ID**	**Bacterial species**	**Sequence type type**	**MIC (μg/ml)**												***rmpA2***	***bla*_*KPC*__–__2_**	**Other acquired virulence and resistance genes**	**Conjugation efficiency**
			**CAZ**	**CTX**	**IPM**	**MEM**	**ETP**	**AK**	**CIP**	**PB**	**ATM**	**TIG**	**CZA**	**FDC**				
SH12	*K. pneumoniae*	11	>128	>128	>128	>128	>128	>128	128	0.5	>128	0.5	4/4	16	+	+	*iucA*, *qnrB4*, *bla*_*DHA*__–__1_, *sul1*, *msr(E)*, *mph(E)*, *bla*_*TEM*__–1B_, *aac(3)-IId*, *bla*_*SHV*__–__12_, *mph(A)*, *bla*_*CTX*__–__*M*__–__65_, *bla*_*TEM*__–1B_, *fosA3*, *rmtB*	NA
J53	*E. coli*	NA	0.25	0.25	0.5	0.03	0.03	4	0.008	0.5	0.125	0.25	0.125/4	0.25	–	–	–	6.00E–06
J53-C1	*E. coli*	NA	16	32	0.5	0.03	0.03	4	0.008	0.5	16	0.25	0.125/4	2	+	–	*iucA*, *qnrB4*, *bla*_*DHA*__–__1_, *sul1*, *msr(E)*, *mph(E)*, *bla*_*TEM*__–1B_, *aac(3)-IId*, *bla*_*SHV*__–__12_, *mph(A)*	2.00E–06^a^
J53-C2	*E. coli*	NA	16	32	0.5	0.03	0.03	4	0.008	0.5	16	0.25	0.125/4	2	+	–	*iucA*, *qnrB4*, *bla*_*DHA*__–__1_, *sul1*, *msr(E)*, *mph(E)*, *bla*_*TEM*__–1B_, *aac(3)-IId*, *bla*_*SHV*__–__12_, *mph(A)*	2.00E–06^a^
J53-C3	*E. coli*	NA	16	32	4	4	4	>128	0.008	0.5	16	0.25	0.125/4	2	+	+	*iucA*, *qnrB4*, *bla*_*DHA*__–__1_, *sul1*, *msr(E)*, *mph(E)*, *bla*_*TEM*__–1B_, *aac(3)-IId*, *bla*_*SHV*__–__12_, *mph(A)*, *bla*_*CTX*__–__*M*__–__65_, *bla*_*TEM*__–1B_, *fosA3*, *rmtB*	1.00E–06
J53-C4	*E. coli*	NA	16	32	0.5	0.03	0.03	4	0.008	0.5	16	0.25	0.125/4	2	+	–	*iucA*, *qnrB4*, *bla*_*DHA*__–__1_, *sul1*, *msr(E)*, *mph(E)*, *bla*_*TEM*__–1B_, *aac(3)-IId*, *bla*_*SHV*__–__12_, *mph(A)*	2.00E–06^a^
J53-C5	*E. coli*	NA	16	32	4	4	4	>128	0.008	0.5	16	0.25	0.125/4	0.25	–	+	*bla*_*CTX*__–__*M*__–__65_, *bla*_*TEM*__–1B_, *fosA3*, *rmtB*	3.00E–06
J53-C6	*E. coli*	NA	16	32	0.5	0.03	0.03	4	0.008	0.5	16	0.25	0.125/4	2	+	–	*iucA*, *qnrB4*, *bla*_*DHA*__–__1_, *sul1*, *msr(E)*, *mph(E)*, *bla*_*TEM*__–1B_, *aac(3)-IId*, *bla*_*SHV*__–__12_, *mph(A)*	2.00E–06^a^
PM23	*K. pneumoniae*	15	0.5	0.06	0.5	0.25	0.25	2	>128	0.5	0.25	0.5	0.5/4	0.25	–	–	–	NA
PM23-C1	*K. pneumoniae*	15	64	128	0.5	0.25	0.25	2	>128	0.5	>128	0.5	1/4	16	+	–	*iucA*, *qnrB4*, *bla*_*DHA*__–__1_, *sul1*, *msr(E)*, *mph(E)*, *bla*_*TEM*__–1B_, *aac(3)-IId*, *bla*_*SHV*__–__12_, *mph(A)*	1.14E–08
PM23-C3-1	*K. pneumoniae*	15	64	128	0.5	0.25	0.25	2	>128	0.5	>128	0.5	1/4	16	+	–	*iucA*, *qnrB4*, *bla*_*DHA*__–__1_, *sul1*, *msr(E)*, *mph(E)*, *bla*_*TEM*__–1B_, *aac(3)-IId*, *bla*_*SHV*__–__12_, *mph(A)*	1.48E–08
PM23-C3-2	*K. pneumoniae*	15	>128	>128	>128	>128	>128	>128	>128	0.5	>128	0.5	1/4	16	+	+	*iucA*, *qnrB4*, *bla*_*DHA*__–__1_, *sul1*, *msr(E)*, *mph(E)*, *bla*_*TEM*__–1B_, *aac(3)-IId*, *bla*_*SHV*__–__12_, *mph(A)*, *bla*_*CTX*__–__*M*__–__65_, *bla*_*TEM*__–1B_, *fosA3*, *rmtB*	2.96E-10
25922	*E. coli*	NA	0.25	0.06	0.25	0.016	0.03	4	0.008	0.5	0.25	0.25	0.125/4	0.25	NA	NA	NA	NA

*CAZ, ceftazidime; CTX, cefotaxime; IPM, imipenem; MEM, meropenem; ETP, ertapenem; AK, amikacin; CIP, ciprofloxacin; PB, polymyxin B; ATM, aztreonam; TIG, tigecycline; CZA, ceftazidime-avibactam; FDC, cefiderocol; NA, Not available.*

*^a^Conjugation efficiency for J53-C1, J53-C2, J53-C4, and J53-C6 is the same.*

Strain SH12 was then subjected to whole-genome sequencing using both the 150-bp paired-end Illumina NextSeq 500 platform and the long-read Oxford Nanopore Technologies MinION platform. The genome size of strain SH12 was found to be 5,922,385 bp, including a 5.19-Mb chromosome and six plasmids with size of 284,671 bp (IncFIB/IncHI1B), 167,468 bp (IncFII/IncR), 10,060 bp (ColRNAI), 6,135 bp (IncQ1), 5,596 bp (ColRNAI), and 3,138 bp, respectively. Strain SH12 was found to belong to ST11 based on MLST, KL47 serotype based on capsular typing, and OL101 O-antigen type. The 6,135-bp IncQ1 plasmid was designated pSH12_4 which carried *ermT* gene coding for macrolide resistance. The chromosome of strain SH12 also harbored *bla*_*SHV*__–__11_ and *fosA* genes.

**FIGURE 1:**
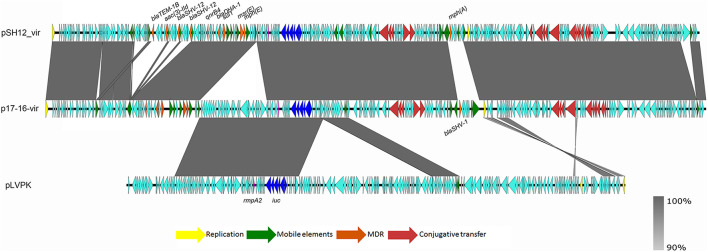
Alignment of pSH12_Vir and plasmids with similar structures. Plasmid pSH12_Vir was an IncFIB/IncHI1B plasmid with several mosaic MDR regions and virulence factors. Genetic regions associated with virulence and resistance are highlighted.

**FIGURE 2:**
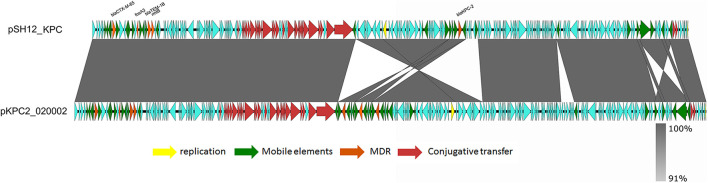
Alignment of pSH12_KPC and plasmid with similar structures. Plasmid pSH12_KPC was highly similar (98% coverage and 99% identity) to plasmid pKPC2_020002 (GenBank accession no. CP028541.2) recovered from a *K. pneumoniae* strain.

Strain SH12 harbored a number of virulence factors, including the regulator of mucoid phenotype 2 (*rmpA2*), aerobactin (*iucABCD*,*iutA*), and yersiniabactin (*ybt* 9; ICEKp3). However, the RmpA2 was found to be truncated at position 126 (nucleotide C^301^A&GGG^284–286^ delete&A^353^ delete), which might cause the negative string test. The *rmpA2* and *iucABCDiutA* genes were found to be located in a 284,671-bp IncFIB/IncHI1B plasmid, designated as pSH12_Vir (Figure 1). Using Blast against the National Center for Biotechnology Information (NCBI) database, this plasmid was found to show highest similarity (90% coverage and 99% identity) to plasmid p17-16-vir (GenBank accession no. MK191024.1), a 290,451-bp IncFIB/IncHI1B plasmid recovered from a *K. pneumoniae* strain isolated from sputum in China. Plasmid p17-16-vir was found to have a 66-kbp insertion from virulence plasmid pLVPK, including the tellurium resistance cluster, *rmpA2* and *iucABCDiutA* genes (Figure 1 and Table 2). Plasmid pSH12_Vir was found to reserve 35 kbp of this fragment, including *rmpA2* and *iucABCDiutA* genes, with the tellurium resistance cluster excluded. These genetic changes indicated the adaption of virulence plasmids with *K. pneumoniae* strains during the transmission process. These virulence plasmids lacked virulence genes *rmpA* and *iroBCDN* when compared to typical virulence plasmid pLVPK, which may affect the virulence potentials of these CRKP strains. However, 96.2% of the virulence plasmids in the GenBank carried *iucA*, while only 46.8% carried *iroB* (Tian et al., 2021). The virulence effects and any benefits of the *iro* deletion remains to be studied. There were also several mobile genetic elements with antimicrobial resistance (AMR) genes found in pSH12_Vir, including a *qnrB4-bla*_*DHA*__–__1_*-sul1* integron and an IS*5-msr(E)-mph(E)-*IS*Kpn21* region which was similar to plasmid p17-16-vir. Other AMR regions were an IS*26-mer-*IS*5075-bla*_*TEM*__–1B_*-*IS*Cfr1-aac(3)-IId-*IS*26-bla*_*SHV*__–__12_*-*IS*26-bla*_*SHV*__–__12_*-*IS*26* region and an IS*Kpn28-mph(A)-*IS*6100-*IS*5075* region. Such virulence-resistance hybrid plasmids have been reported recently, which can be generated by integrating the AMR gene-bearing DNA fragment into the virulence plasmid backbone (Zhang et al., 2016; Shen et al., 2019), as well as integrating the virulence-bearing DNA fragment into the AMR plasmid (Lam et al., 2019; Turton et al., 2019). Strain SH12 was also shown to harbor the carbapenemase gene *bla*_*KPC*__–__2_, which was found to be located in a NTE_*KPC*_-Ib transposon in a 167,468-bp IncFII/IncR plasmid, pSH12_KPC (Figure 2). Plasmid pSH12_KPC was highly similar (98% coverage and 99% identity) to the 177,516-bp IncFII/IncR plasmid pKPC2_020002 (GenBank accession no. CP028541.2) recovered from *K. pneumoniae* strain WCHKP2 (Figure 2 and Table 2). Plasmid pSH12_KPC was also found to harbor *bla*_*CTX*__–__*M*__–__65_, *bla*_*TEM*__–1B_, *fosA3*, and *rmtB* resistance genes.

**TABLE 2:** Comparison of plasmids pSH12_Vir and pSH12_KPC with similar plasmids in the GenBank database.

**Plasmid ID**	**Plasmid type**	**Size (bp)**	**Virulence genes**	**AMR genes**	***tra* genes**	**Genbank No.**
pSH12_Vir	IncFIB/IncHI1B	284,671	*rmpA2*, *iucABCDiutA*	*qnrB4*, *bla*_*DHA*__–__1_, *sul1*, *msr(E)*, *mph(E)*, *bla*_*TEM*__–1B_, *aac(3)-IId*, *bla*_*SHV*__–__12_, *mph(A)*	+	CP040834
p17-16-vir	IncFIB/IncHI1B	290,451	*rmpA2*, *iucABCDiutA*, tellurium resistance genes	*qnrB4*, *bla*_*DHA*__–__1_, *sul1*, *msr(E)*, *mph(E)*, *bla*_*SHV*__–__1_	+	MK191024
pSH12_KPC	IncFII/IncR	167,468	–	*bla*_*KPC*__–__2_, *bla*_*CTX*__–__*M*__–__65_, *bla*_*TEM*__–1B_, *fosA3*, *rmtB*	+	CP040835
pKPC2_020002	IncFII/IncR	177,516	–	*bla*_*KPC*__–__2_, *bla*_*CTX*__–__*M*__–__65_, *bla*_*TEM*__–1B_, *fosA3*, *rmtB*	+	CP028541

**FIGURE 3:**
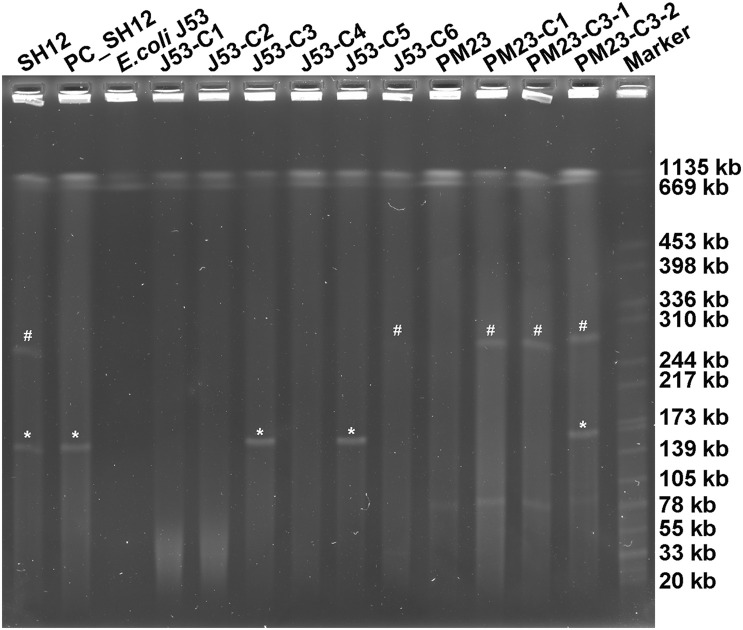
S1-PFGE of strain SH12 and its transconjugants. Transconjugants J53-C1, J53-C2, J53-C4, and J53-C6 were found to receive virulence plasmid only; J53-C5 was found to receive the KPC plasmid only; J53-C3 was found to receive both plasmids. Transconjugant PM23-C1 was generated using J53-C1 as donor. Transconjugants PM23-C3-1 and PM23-C3-2 were generated using J53-C3 as donor. “#” denotes virulence plasmid pSH12_Vir, and “*” denotes KPC plasmid pSH12_KPC.

More importantly, carrying the self-transmission systems, mosaic multidrug resistance (MDR)-virulence plasmids could be transferred into hvKP or cKP strains, resulting in MDR-HvKP strains (Tang et al., 2020). As plasmid pSH12_Vir and pSH12_KPC were both shown to harbor conjugative transfer genes, the transferability of these two plasmids were determined by conjugation. Firstly, *E. coli* strain J53 was used as recipient strain. Both the virulence and KPC plasmid were found to be able to transfer to strain J53 (Table 1 and Figure 3). Transconjugants J53-C1 showed resistance to β-lactam antibiotics, ceftazidime, cefotaxime, and aztreonam and was positive for *rmpA2*, a marker gene of virulence plasmid pSH12_Vir by PCR, indicating that plasmid pSH12_Vir was acquired. Transconjugants J53-C5 showed resistance to β-lactam antibiotics, including carbapenem and amikacin, and was positive for *bla*_*KPC*__–__2_, a marker gene of the KPC plasmid pSH12_KPC by PCR, indicating that plasmid pSH12_KPC was acquired. Interestingly, transconjugant J53-C3 was positive for both *rmpA2* and *bla*_*KPC*__–__2_ genes, indicating that transconjugants received these two plasmids simultaneously. The KPC plasmid was confirmed in the according transconjugants J53-C3 and J53-C5 by S1-PFGE (Figure 3). The virulence plasmid in the *E. coli* transconjugants was hardly observed even though several colonies were tried, namely, J53-C1, J53-C2, J53-C4, and J53-C6, which were identified as acquiring virulence plasmid pSH12_Vir by MIC and PCR assays; a weak band was observed of transconjugant J53-C6 (Figure 3). This might be caused by low copy numbers of this plasmid in *E. coli*. To further determine the transferability of the virulence plasmid, transconjugant strain J53-C1 and J53-C3 were used as donors, respectively, and ciprofloxacin-resistant *K. pneumoniae* strain PM23 was used as recipient. It was found that virulence plasmid pSH12_Vir could be transferred to *K. pneumoniae* strain PM23 alone as well as together with pSH12_KPC from those *E. coli* transconjugants. When J53-C3 was used as donor, transconjugant PM23-C3-1 was found to receive only the virulence plasmid; transconjugant PM23-C3-2 was found to receive both the virulence and KPC plasmid (Table 1 and Figure 3). Interestingly, strain SH12 was resistant to cefiderocol with a MIC of 16 μg/ml, and strains that received plasmid pSH12_Vir also exhibited increased resistance to cefiderocol (Table 1), indicating possible resistance determinants on plasmid pSH12_Vir. Limited data of cefiderocol resistance mechanisms are currently available, and deficiencies in the iron transporter systems (Ito et al., 2018), serine β-lactamases, and metallo-β-lactamases (MBLs) (Kohira et al., 2020) might play roles in conferring resistance to cefiderocol. Thus, serine β-lactamases, DHA-1 and TEM-1B, encoded by plasmid pSH12_Vir, may play a role in mediating resistance to cefiderocol, which needs further confirmation.

**FIGURE 4:**
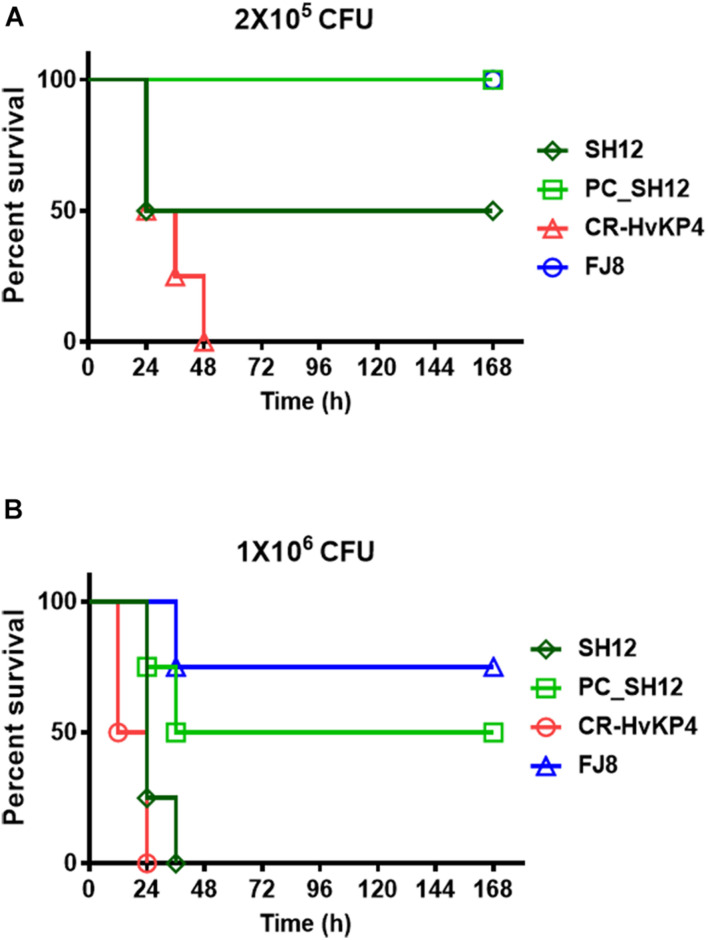
Virulence level of different bacterial strains as depicted in mouse infection model. Survival of mice (*n* = 8) infected by 2 × 10^5^ CFU **(A)** and 1 × 10^6^ CFU **(B)** of each *K. pneumoniae* strain at 168 h. The test strains included *K. pneumoniae* strain SH12, its virulence plasmid cured strain PC_SH12, CR-HvKP strain CR-HvKP4 (hypervirulence control), and the classic CRKP strain FJ8 (low virulence control).

The virulence level of strain SH12 was tested in male ICR mouse. Upon being infected for 1 week at an inoculum of 1 × 10^6^ CFU, survival of mice was 0% with strain CR-HvKP4, 0% with SH12, and 75% with strain FJ8 (Figure 4B). While at an inoculum of 2 × 10^5^ CFU, survival of mice was 0% with strain CR-HvKP4, 50% with SH12, and 100% with strain FJ8 (Figure 4A). These data all showed that strain SH12 possessed moderate virulence potential. To determine virulence contribution of the virulence plasmid to strain SH12, plasmid curing was performed. The plasmid-cured strain, designated as PC_SH12, was then subjected to mice infection model. Upon being infected for 1 week at inoculums of 1 × 10^6^ and 2 × 10^5^ CFU, survival of mice was 50 and 100% with strain PC_SH12, respectively, indicating that virulence plasmid pSH12_Vir played an important role in determining virulence of strain SH12 (Figure 4).

## Conclusion

In conclusion, this study characterized a CRKP strain which harbored a conjugative virulence plasmid. This virulence plasmid was generated by integration of a 35-kbp fragment containing *rmpA2* gene and aerobactin encoding genes from a pLVPK-like virulence plasmid into a resistance plasmid. Generation of conjugative plasmid simultaneously carrying virulence and resistance-encoding genes will increase the spread of these elements among pathogens. This virulence plasmid was found to be able to transfer together with the KPC plasmid, which may promote prevalence of CR-HvKP infections. Findings in this work therefore provide important insight into the evolution of virulence-encoding elements during transmission in *K. pneumoniae* strains.

## Data Availability Statement

The datasets presented in this study can be found in online repositories. The names of the repository/repositories and accession number(s) can be found below: https://www.ncbi.nlm.nih.gov/genbank/, CP040833-CP040839.

## Ethics Statement

The animal study was reviewed and approved by the Animal Research Ethics Sub-Committee, City University of Hong Kong.

## Author Contributions

XY performed the experiment and drafted the manuscript. ND helped with the bioinformatic analysis. XL and CY performed the S1-PFGE. LY performed the DNA sequencing. EC edited the manuscript and contributed to experimental design. RZ and SC designed, supervised the study, and interpreted the data. SC wrote the manuscript. All authors contributed to the article and approved the submitted version.

## Conflict of Interest

The authors declare that the research was conducted in the absence of any commercial or financial relationships that could be construed as a potential conflict of interest.

## Publisher’s Note

All claims expressed in this article are solely those of the authors and do not necessarily represent those of their affiliated organizations, or those of the publisher, the editors and the reviewers. Any product that may be evaluated in this article, or claim that may be made by its manufacturer, is not guaranteed or endorsed by the publisher.

## Footnotes

1
http://bigsdb.pasteur.fr/klebsiella/klebsiella.html

2
http://www.genomicepidemiology.org/

3
https://www-is.biotoul.fr/index.php
